# T Cells Enhance Stem-Like Properties and Conditional Malignancy in Gliomas

**DOI:** 10.1371/journal.pone.0010974

**Published:** 2010-06-07

**Authors:** Dwain K. Irvin, Emmanuel Jouanneau, Gretchen Duvall, Xiao-xue Zhang, Yuying Zhai, Danielle Sarayba, Akop Seksenyan, Akanksha Panwar, Keith L. Black, Christopher J. Wheeler

**Affiliations:** Department of Neurosurgery, Maxine Dunitz Neurosurgical Institute, Cedars-Sinai Medical Center, Los Angeles, California, United States of America; City of Hope Medical Center and Beckman Research Institute, United States of America

## Abstract

**Background:**

Small populations of highly tumorigenic stem-like cells (cancer stem cells; CSCs) can exist within, and uniquely regenerate cancers including malignant brain tumors (gliomas). Many aspects of glioma CSCs (GSCs), however, have been characterized in non-physiological settings.

**Methods:**

We found gene expression similarity superiorly defined glioma “stemness”, and revealed that GSC similarity increased with lower tumor grade. Using this method, we examined stemness in human grade IV gliomas (GBM) before and after dendritic cell (DC) vaccine therapy. This was followed by gene expression, phenotypic and functional analysis of murine GL26 tumors recovered from nude, wild-type, or DC-vaccinated host brains.

**Results:**

GSC similarity was specifically increased in post-vaccine GBMs, and correlated best to vaccine-altered gene expression and endogenous anti-tumor T cell activity. GL26 analysis confirmed immune alterations, specific acquisition of stem cell markers, specifically enhanced sensitivity to anti-stem drug (cyclopamine), and enhanced tumorigenicity in wild-type hosts, in tumors in proportion to anti-tumor T cell activity. Nevertheless, vaccine-exposed GL26 cells were no more tumorigenic than parental GL26 in T cell-deficient hosts, though they otherwise appeared similar to GSCs enriched by chemotherapy. Finally, vaccine-exposed GBM and GL26 exhibited relatively homogeneous expression of genes expressed in progenitor cells and/or differentiation.

**Conclusions:**

T cell activity represents an inducible physiological process capable of proportionally enriching GSCs in human and mouse gliomas. Stem-like gliomas enriched by strong T cell activity, however, may differ from other GSCs in that their stem-like properties may be disassociated from increased tumor malignancy and heterogeneity under specific host immune conditions.

## Introduction

The cancer stem cell (CSC) hypothesis posits that neoplastic clones are exclusively maintained by a small fraction of cells with stem cell properties in many tumors including glioblastoma multiforme (GBM), the most malignant primary brain tumor (glioma) [1]. CSCs are rare within GBM, but are classically enriched by selection of stem markers such as CD133, or by neurosphere formation [1], [2]. Classical glioma CSCs (GSCs) regenerate both themselves and more differentiated tumor progeny [3], but GSC differentiation results in decreased tumorigenicity [4], [5], [6], [7], [8], [9]. Thus, destruction or differentiation of CSCs is thought necessary and perhaps sufficient to effectively treat tumors such as GBM [10].

Key aspects of GBM malignancy, however, are not easily integrated into the CSC hypothesis. For example, despite their higher tumorigenicity, CSCs are more prominent in pediatric brain tumors such as medulloblastoma and ependymoma than in GBM [1],[2]. In this context, medulloblastomas in particular can be highly malignant, as evidenced by their assignment of WHO grade IV, but their overall, progression-free, and 5-year survival rates are comparable to those of ependymoma, and far exceed those of GBM [11] [12] [13]. Thus, CSCs are more prominent in brain tumors with substantially lower malignancy than GBM. In addition, cytolytic treatments such as irradiation and chemotherapy that enrich GSCs [14], [15], clinically benefit brain tumor patients [16], [17]. This could be due to a primary influence of GSCs on tumor recurrence, rather than on overall tumor progression, although GSC-enriching therapies appear to delay recurrences to some extent as well [16], [17]. Moreover, the effectiveness of anti-CSC therapy in treating CSC-rare tumors has not been demonstrated, although it may effectively treat tumors with sizable CSC subpopulations [18]. Finally, classical CSCs appear to exacerbate malignancy within discrete glioma subcategories [19], implicating modification of stem-associated malignancy by independent tumor properties.

Since many aspects of CSCs have been characterized in non-physiological *in vitro* or *in vivo* systems, further resolution of the relationship of CSCs to tumor malignancy may hinge on identifying inducible physiological processes that enhance stem-like properties. As CSCs are enriched by cytolytic therapy, we examined whether cytolytic T cell activity [20], [21] might represent one such process. We found that T cell activity enhances most genetic and functional stem-like properties within gliomas, but fails to unconditionally enhance either tumorigenicity or heterogeneous gene expression, thereby providing further clarification on the role of stem-like tumors in glioma malignancy and diversity.

## Methods

### Patients

All CSMC patients participating in this study provided written informed consent for gene profiling, vaccination (where appropriate), and all associated analyses prior to surgery. Human investigations were performed after approval by the Cedars-Sinai Medical Center institutional review board and in accord with an assurance filed with and approved by the U.S. Department of Health and Human Services. Data from non-CSMC patients was acquired from publications and/or public databases, and was not linked to personal information or identifiers. Vaccination of the 6 patients whose tumors are included in microarray analyses here was performed as previously described in the literature under phase I [22], or phase II [23] tumor lysate/DC trials (vaccine trial #s 1 and 2, respectively; trial #2 was a phase II continuation of trial #1, and employed identical manufacturing and monitoring protocols), or according to an unpublished phase I vaccine trial (vaccine trial #3), as shown in Table 1.

**Table 1:** 

Fig. S3 designation	Trial #	Total # pts	Reference & pt #	NCI Registry #
Pr1, Po1	2	34	[23], pt #5	NCT00576537
Pr2, Po2	1	14	[22], pt # 3	NA
Pr3, Po3	3	5	NA	NA
Pr4, Po4	1	14	[22], pt #6	NA
Pr5, Po5	2	34	[23], pt #20	NCT00576537
Pr6, Po6	1	34	[22], pt #14	NA

Trial #s 1 and 3 completed enrollment and treatment prior to 2005, and do not have NCI registry numbers. Design & objectives of each trial were to assess safety, as well as immunological and clinical responses, after vaccine administration to: 12 recurrent and 2 newly diagnosed high-grade glioma patients (10 GBM, 4 anaplastic astrocytoma; trial #1) [22]; 21 recurrent and 11 newly-diagnosed high-grade glioma patients (all GBM; trial #2) [23]; and 5 recurrent GBM patients (trial #3, unpublished) as detailed in their respective protocols (included as supporting documents). Immunological responses and clinical outcomes have already been reported for phase I and phase II DC/tumor lysate vaccine trials [22], [23].

For DC vaccination trials, patients were administered 3 vaccines, 2 weeks apart, followed in some cases by a third vaccine 6 weeks later, of up to 10^7^ autologous tumor lysate-pulsed dendritic cells (differentiated from monocytes using IL-4 and GM-CSF) following irradiation therapy as described [22], [23]. Informed consent was obtained from all patients prior to enrollment into vaccination trials (enrollment 1999–2005). For all trials, the primary safety endpoint was the number of Grade 3 or 4 toxicities, and was evaluated in all enrolled patients. Primary efficacy endpoints were time to survival (TTS) and time to tumor progression (TTP). TTS was evaluated from the date of surgery immediately preceding vaccination to the date of death or last contact (if still living). TTP was evaluated from the same initial surgery date used for TTS, to date of progression on MRI (approximately 25% increase in tumor volume), provided progression was verified either histologically or in serial MRI scans (including routine FLAIR, gadolinium-enhanced, and perfusion-weighted MRIs). All diagnostic pathology and scans were subjected to central tumor board review and consensus.

Post-vaccine immune responsiveness was the primary immunological endpoint. Vaccinated patients were corticosteroid-free during all blood collections and vaccinations. Each of up to 4 vaccines consisted of 900 µg autologous tumor lysate/10-40×10^6^ autologous DC. Vaccination started approximately 15 weeks post-surgery. Serial MRI scans were performed every 2–3 months, continuously monitored until late-2006. Tumor lysates were derived from a single surgical tumor resection immediately preceding vaccination. No effect of sex or any of the major ethnic groups on safety, immunological, or efficacy endpoints was detected. All vaccine patients from whom surgically resected tumor tissue was available before and after vaccination, and that yielded RNA of sufficiently quality and quantity for microarray analysis, were analyzed. This comprised 10–20% of all patients enrolled on any given vaccine trial as summarized above. Primary immunological endpoints are presented here only for GBM patients.

### RNA isolation and microarray analysis

At the time of resection, tumors were examined by a neuropathologist and dissected into two portions, one for tissue diagnosis by histopathology and the other for RNA extraction. This procedure was done within 15 minutes of surgical resection while the tissue was kept on ice. The RNA extraction portion was then snap frozen in liquid nitrogen and stored at −80°C. Most of the tumor samples were about 0.5 cm in diameter. Total RNA from tissue was extracted as previously described [24]. Only samples with sufficient quality and quantity of RNA were further analyzed. 10 µg of total RNA was used to synthesize double stranded cDNA using Superscript Choice (Invitrogen, Carlsbad, California, USA). Biotin-labeled antisense cRNA was synthesized by in vitro transcription using the ENZO BioArray HighYield kit (Enzo Diagnostics, Farmingdale, New York, USA). 20 µg cRNA was chemically fragmented and was hybridized to Affymetrix HG-U133 Plus 2 (human) and MG-430 Plus 2 (mouse) GeneChip arrays (Affymetrix, Santa Clara, California, USA). The quality, yield, and size distribution of total RNA, labeled transcripts, and fragmented cRNA were estimated by spectrophotometric analysis at 260 and 280 nm and electrophoresis on RNA 6000 Nano-LabChips (Agilent Technologies, Palo Alto, California, USA). Arrays were washed, stained with streptavidin-phycoerythrin, and scanned to generate image files. Array data was acquired as MAS5-normalized (intra-sample) values. 1583 human probe-sets exhibiting significant alteration in GBM tissue after vaccination (>1.3-fold change post-vaccine relative to pre-vaccine; p<0.05) were identified using dChip 2006 software (Table S1). Data was normalized between samples in dChip or GeneSpring software, and subjected to Principal Component analysis (PCA) using the 1583 probesets.

### DC vaccination of mice

Wild-type C57BL/6J mice were vaccinated 3 and 7 days post-tumor implantation with 10^7^ cultured DC2.4 cells that had been pulsed 2 hr with GL26 cell lysate (150 µg/ml) injected subcutaneously on the flank. CTL assay and flow cytometric stainings of splenocytes and/or excised brain tumors was performed on selected terminally symptomatic mice, and exhibited consistent evidence of anti-tumor T cell function or expansion, respectively.

### Microarray, Principal Component Analysis, and Heirarchical Clustering

Vaccine altered gene lists in GL26 were generated in dChip 2006 and included probesets with a significant (> (*P*<0.05 by one-tailed T-test and/or ANOVA) >1.5-fold change in GL26 tumors recovered from nude vs. vaccinated C57BL/6J (probesets, gene titles, and post-/pre-vaccine expression values available in Tables S1 and S2). Immune Modulator gene lists include human or mouse IL-1,IL-1Rs, IL-6, IL-10, IL-23; prostaglandin E synthase; Ccl5; Stat3; Tlr genes; MIG; MIP-1a, b; MCP1,2,3; CD70; CD274; MHC-Ia and MHC–II genes; ST3Gal-I, ST3Gal-II; Fas, FasL; and glucoceramide synthase. Sonic Hedgehog and EGFR gene lists include genes regulated by either Sonic Hedgehog and/or EGFR, according to published sources (probesets and gene titles available in Tables S1 and S2). Progenitor/Differentiation gene lists were adapted from Superarray.com website (probesets and gene titles available in Tables S1 and S2). Principal Component Analysis was performed using GeneSpring GX 7.3, and Heirarchical Clustering performed in dChip 2006.

### Pathway identification

The differentially expressed probe sets were overlaid on a cellular pathway map in the Ingenuity Pathway Analysis using resource database Knowledge Base (Winter 04 Release containing 20,000 genes). The resulting networks are represented in table and graphic format.

### Real-time quantitative PCR

Total RNA was extracted from GL26 tumor cells recovered from 3 nude, 3 C57BL/6J, and 3 DC-vaccinated C57BL/6J mouse brains using Trizol (Invitrogen). RNA quality was confirmed by spectrometry and gel electrophoresis. cDNA was prepared from 2 ug total RNA using random hexamers and oligo DT (BioRad, Hercules, CA). Serially diluted pooled cDNA was amplified with specific primers to assess reaction efficiency, and tumor samples were amplified in triplicate and detected with SybrGreen. Mouse (m) GLI1 forward 5′-ATC TCT CTT TCC TCC TCC TCC-3′, GLI1 reverse 5′-CGA GGC TGG CAT CAG AA-3′; mSHH forward 5′-GCT CGC CTG GCT GTG GA-3′, reverse 5′-CGC CAC GGA GTT CTC TGC TTT-3′; mN-MYC forward 5′- GGA TGA TCT GCA AGA ACC CAG-3′, reverse 5′- GTC ATC TTC GTC CGG GTA GAA-3′; mEGFR forward 5′- AAT CCC AGG ACC AAC TAT GGC AGC-3′, reverse 5′- GAG GCA AAC TTC TGT TCC AAT GG-3′; mCD133 forward 5′- TCA GAC CTG GAT GGC ATC GG-3′, reverse 5′- CGC CAT GGC CTT AAT CTC TTC G-3′; mGFAP forward 5′- GCC ACG CTT CTC CTT GTC TCG-3′, reverse 5′- GCC CGT GTC TCC TTG AAG CC-3′; mGAPDH forward 5′- GGC CTT CCG TGT TCC TAC-3′, reverse 5′- TGT CAT CAT ACT TGG CAG GTT-3′. Threshold cycle was calculated relative to an arbitrary internal control according to the method of Vandesompele [25]. Expression values were normalized relative to simultaneously amplified GAPDH in all samples, plotted with standard error bars, and differences assessed by ANOVA.

### CTL assays

Target cell killing by native T cells was according to cytotoxicity detection kit instructions (LDH, Roche Diagnostics GmbH, Mannheim, Germany). Briefly, splenocyte effector cells from DC-vaccinated or non-vaccinated GL26-bearing mice were titrated in assay medium in sterile 96-well tissue culture plates by serial dilution (100 µl/well). After washing, GL26 cell suspension (5×10^5^ cell/ml) was added at 3.3∶1, 10∶1, and 30∶1 effector:target (E:T) ratios in triplicate, with additional wells prepared for negative, positive, and splenocyte controls followed by incubation of cells, 37°C, 5% CO2, 90% humidity) for 6 hours. Thereafter 100 µl/well supernatant was carefully removed and transferred into corresponding wells of an optically clear 96-well flat bottom microplate. 100 µl reaction mixture was added to each well and incubated 25 min, 25°C in dark. Absorbance at 490 or 492 nm was measured using ELISA reader. Cytotoxicity was calculated as [(splenocytes GL26 cell mix - splenocyte control)-negative control]/[positive control - negative control] x 100, and plotted with standard error bars. Identical methods were used to determine antigen-independent killing sensitivity of GL26, GL26B6, GL2B6V, GL26Nu tumor cells, except that 5×10^5^ cell/ml tumor cells were co-incubated with HTB-157.7 cells, a non-adherent H-2K^b^-reactive T cell hybridoma, as effectors at 10∶1, 20∶1, and 40∶1 E:T ratios in triplicate for 12–15 hours prior to reading absorbance.

### Tissue histology, immunofluorescence and flow cytometry

#### Serial coronal sections

16 µm sections of brains of nude and DC2.4-vaccinated C57BL/6J mice with GL26 tumor were cut on a cryostat, and mounted to gelatin-coated slides. For H&E staining & GFP analysis, frozen glioma sections (14 µm) were fixed with acetone. Endogenous peroxidase activity was eliminated with 0.3% H_2_O_2_/PBS before either visualizing GFP fluorescence without additional staining, or staining with eosin and counterstain with hematoxylin. Slides were visualizated at 10X magnification.

#### Immunofluorescence staining

sections were fixed in 4% paraformaldehyde and incubated with primary antibodies 1 hr at room temp: rat anti-CD133/IgG1 (Chemicon International; USA., 1∶ 100); rabbit anti-Sox-2/IgG (Chemicon International; 1∶ 100); rabbit anti-GFAP/IgG (Sigma-Aldrich; 1∶ 100), and mouse anti-Nestin Clone Rat 401/IgG1 (Chemicon International; USA., 1∶ 100) diluted in 5% normal goat serum, 0.1% Triton-X. Fluorescein- or Texas red-conjugated anti-rat, anti-rabbit, or anti-mouse secondary antibody were applied, and images of tumors from nude and vaccinated mouse brains were obtained from a Zeiss axiophot epifluorescence microscope (Carl Zeiss MicroImaging, Inc., Thornwood, NY) at 20X using identical camera settings.

#### Flow cytometry

GL26 tumor cells recovered from 3 nude, 3 C57BL/6J, and 3 DC-vaccinated C57BL/6J mouse brains were stained with the following Ab: Ki67: Clone B56 (BD Pharmingen, San Jose, CA.) @ 1∶25; rabbit anti-rodent GFAP Polyclonal (Sigma, St. Louis, MO.) @ 1∶100; rat anti-mouse Nestin Clone Rat 401 (Chemicon, Temecula, CA.) @ 1∶1000; rat anti-mouse CD133 Clone 13A4 (Chemicon, Temecula, CA.) @ 1∶200; Rabbit anti-rodent SOX-2 Polyclonal (Chemicon, Temecula, CA.) @ (1∶1000); Goat ant-rodent Shh: Polyclonal (R&D Systems, Minneapolis, MN.) @ 1∶250; Goat ant-mouse EGFR: Polyclonal (R&D Systems, Minneapolis, MN.) @ 1∶250. All 1° Abs were incubated with cells 30 min in 2% heat-inactivated FBS ½ (FACS buffer) hr on ice, washed and incubation with appropriate 2° Ab @ 1∶250 in FACS buffer, 30 min on ice. Cells were run on a FacScan II cytometer with equivalent gain, gates for positive staining set according to negative (2° Ab only) controls, and analysis performed with Cell Quest Software (Stanford, CA.)

### Drug Sensitivity

Fresh stock solutions of commercially obtained drugs (5 mg/ml Erlotinib in DMSO; Genentech, S. San Francisco, or 1 mg/250 ul Cyclopamine in 95% ETOH; Toronto Research Chemicals, Inc, ON, Canada) were prepared for each assay. GL26nu, GL26B6 and GL26B6V cells (recovered as in microarray methods above) were cultured at 100,000 per well in 1 mL complete RPMI. Erlotinib or cyclopamine was added at the indicated concentrations to growth medium without serum, and cultured 24 hr. Cell suspensions were collected and viable cell numbers determined on a cell counter (Z2 Coulter Corp, Miami, FL.)

### Tumor Cell Implantation in Mice

Female C57BL/6 (Jackson Labs) and nude mice (Foxn1; Harlan, Inc.) were housed in a pathogen–free vivarium and used on protocols approved by the Cedars-Sinai Medical Center IACUC according to federal guidelines. The murine (C57BL/6) GL26 glioma cell line, which is highly tumorigenic in syngeneic C57BL/6 mice [26], [27], [28], was obtained with permission from Dr. Henry Brem (Johns Hopkins, Baltimore, MD). GL26 cells were cultured in complete RPMI 1640 medium supplemented with 10% heated-inactivated FBS, 10 mM Hepes, 100 U/ml penicillin, 100 ug/ml streptomycin, and 2 mM L-glutamine (Invitrogen Corp., N.Y., USA). Cultured GL26 glioma cells were harvested by trypsinization, and 50,000 GL26 tumor cells intracranially implanted in 2 ml 1% methylcellulose implanted using a stereotactic rodent frame, with injection 1 mm posterior and 2.5 mm lateral to the junction of the coronal and saggital sutures (bregma), at a depth of 2 mm. GL26 tumors in C57BL/6J mice typically ranged from 15–65 mg. Survival (days from tumor implantation to acquisition of characteristic spectrum of terminal neurological symptoms) was assessed by 2-tailed Mann-Whitney log-rank.

### Tumor retrieval, cell culture and transfection

Primary mouse brain tumor cultures were established from GL26 tumor removed at time of euthanasia after acquisition of terminal neurological symptoms. Tumor tissue was finely minced using sterile scissors, rinsed with dissection medium, and dispersed with trypsin-EDTA. Monolayer cells were plated in T75 flasks (Costar) and culture in DMEM/Ham's F12 (Invitrogen,) supplemented with 10% FBS, L-glutamine, and antibiotics (100 units/ml penicillin and 100 mg/ml streptomycin). Non-adherent cells were washed 12-18 hr post-culture, and thereafter as needed. Adherent cells were grown for 3-8 passages in culture (3–4 passages for GL26nu-1 & -2; 6 passages for GL26B6-1 & -2; 5-6 passages for GL26B6V-1 & -2; 8 passages for all others) RNA was extracted from cells grown to 80% confluence. GL26B6 cells were transfected with pGFP plasmid (Clonetech) using Lipofectin reagent (Invitrogen), followed by selection in medium containing 1.7 mg/ml G418. Uniform expression of GFP by GFP-GL26B6 cells was verified by flow cytometry prior to brain implantation.

GL26 derivative lines (parental GL26, GL26nu, GL26B6, GL26B6V, and GL26B6-GFP) were validated by 100% intracranial tumorigenicity in T cell-deficient hosts at doses of 50,000 cells or less. DC 2.4 cells were validated by flow cytometry against MHCII, CD80 and CD86, and by ability to induce anti-tumor T cell responses by tumor pMHC I tetramer staining (gp100, Trp-2), and/or CD8 + IFN-γ co-staining of effectors.

### Statistical Methods

All statistical methods are indicated for specific assays in their respective figure legends. All in vitro and animal experiments were repeated at least twice (≥3 total repetitions) with similar results.

## Results

### T cell-exposed GBM and GL26 gliomas up-regulate stem-like genes and proteins

Because infallible markers of cancer stem cells have not been described, we first asked whether assessing stem-like properties of GBM tumors by global gene expression similarity could discern GSCs from parental GBMs better than a currently available single marker, CD133. We thus compared the ability of global similarity to an averaged GSC microarray expression profile, and that of CD133 expression, to distinguish GSC lines from native tumors in two separate databases (Fig. S1). Microarray gene expression profile similarity correlated significantly with CD133 expression in both databases (Fig. S1A). Nevertheless, microarray profile similarity effectively distinguished all GSC samples from non-fractionated GBM (Fig. S1B), whereas CD133 expression failed to distinguish any GSCs from GBM, indicating relative superiority in defining CSC lines by microarray profile similarity. Moreover, secondary GBM and grade 3 gliomas exhibited greater microarray similarity to GSCs than did *de novo* GBM (Fig. S1C), whereas CD133 expression is reportedly more prevalent in *de novo* GBM [29]. In fact, microarray data also indicated that CD133 expression was significantly higher in *de novo* than in either secondary GBM or in grade 3 gliomas (Fig. S1C), consistent with previously published findings [1], [2]. Nevertheless, this discrepancy also emphasizes that CD133 expression alone does not accurately identify all GSCs [29], and may not reflect stemness as defined by independent means. This also validated the use of microarray GSC similarity to distinguish stem-like gliomas more faithfully than CD133 expression (Fig. S1 and legend).

We next generated microarray expression profiles from patients' GBM acquired before and after therapeutic dendritic cell (DC) vaccination or before and after standard therapy. We then determined the relatedness of these profiles to those of previously published GBM and GSCs [29]. In addition, GL26 glioma cells were implanted into brains of T cell-deficient (nude), wild-type syngeneic (C57Bl/6; WT), or DC-vaccinated syngeneic mice, and recovered by briefly culturing tumors excised from terminally symptomatic hosts. This recovery yielded GL26nu, GL26B6, and GL26B6V cells from nude, wild-type, and DC vaccinated-wild-type, respectively. We then generated microarray expression profiles from GL26nu, GL26B6, and GL26B6V RNA in parallel with the human studies.

**Figure 1:**
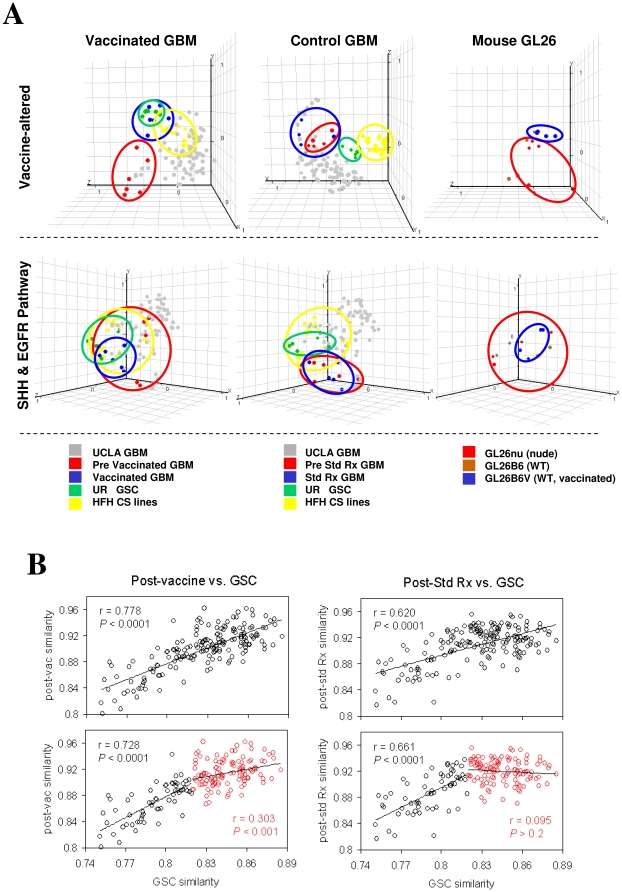
Expression microarray profiles of human GBM and mouse glioma. (A) Principal Component Analyses focused on discrete gene lists were plotted in GeneSpring GX 7.3, and group clusters circled, on: 59 GBMs from UCLA database (“UCLA GBM”; GEO accession #GSE4412), 12 GBMs from 6 patients collected before and after DC vaccination (“vaccinated GBM”; GEO accession #GSE9166); 10 GBMs from 5 patients collected before and after standard radiation and/or chemotherapy (“control GBM”; GEO accession #GSE9166) (red); CD133^-^ and CD133^+^ CSCs from 6 University of Regensberg GBM patients [29] (“UR GSC”; GEO accession #GDS2728) (green); stem cell media-cultured GBM lines from 2 Henry Ford Hospital patients (“HFH CS lines”; GEO accession #GSE4536); murine GL26 glioma samples recovered and cultured ≤8 passages from brains of 5 nude (GL26nu), 4 C57BL/6J (GL26B6) and 4 C57BL/6J mice vaccinated with 10^7^ tumor lysate-pulsed DC2.4 cells 3 and 7d post -tumor implantation (GL26B6V; GEO accession #GSE9166). Post-vaccine GBM uniquely exhibited co-clustering with UCLA glioma progenitors within vaccine altered genes (top row), and similarly constrained expression of SHH and EGFR pathway genes (middle row). Glioma progenitors also exhibited constrained immune modulator gene expression (Fig. S1A). GL26B6V exhibited parallel trends in all analogous gene lists (right column). (B) Primary GBM microarray expression values from 200 Henry Ford Hospital patients (GEO accession #GSE4536) were assessed for similarity to averaged expression values of 6 UCLA glioma CSCs by determining Pearson's coefficients across 54,674 transcripts, and arranged in order of ascending coefficient values. Pearson's coefficients for similarity to the post-vaccine expression profile across all transcripts (averaged from 6 GBM patient samples) were determined, plotted against the first set of coefficients for each patient, and correlation between CSC and vaccine-induced expression profiles calculated using exponential trendlines. This analysis was repeated after subdivision of GBM patients into low (black) and high (red) CSC similarity according to median of relevant Pearson's coefficients (bottom panels).

Principal Component Analysis (PCA) is a decomposition technique that produces a set of expression patterns, or principal components. Linear assemblies of these patterns represent the behavior of all genes in a given sample, characterizing the most abundant themes recurring in many genes of that sample. To determine whether DC vaccination preferentially altered genes distinguishing GSCs from non-stem gliomas, Principal Component Analysis (PCA) was performed using vaccine-altered transcripts (Fig. 1A), Shh and Egfr pathway transcripts (Fig. 1A), or independent immune-modulating gene transcripts, from human GBM and mouse GL26 microarray profiles, and the first three principal components statistically generated and plotted using GeneSpring software (Fig. S2A). This analysis revealed unique co-clustering of post-vaccine but not pre-vaccine GBM to GSCs. Vaccine-exposed GL26 (GL26B6V) similarly clustered away from other GL26 sublines on PCA using analogous sets of transcripts (Fig. 1A). Co-clustering on PCA is an index of relatedness. Hence, these findings suggest increased relatedness of post-vaccine GBM to classical GSCs. Such relatedness was further supported by significantly increased similarity of post-vaccine GBM to GSCs across all transcripts (Fig. S2C).

**Figure 2:**
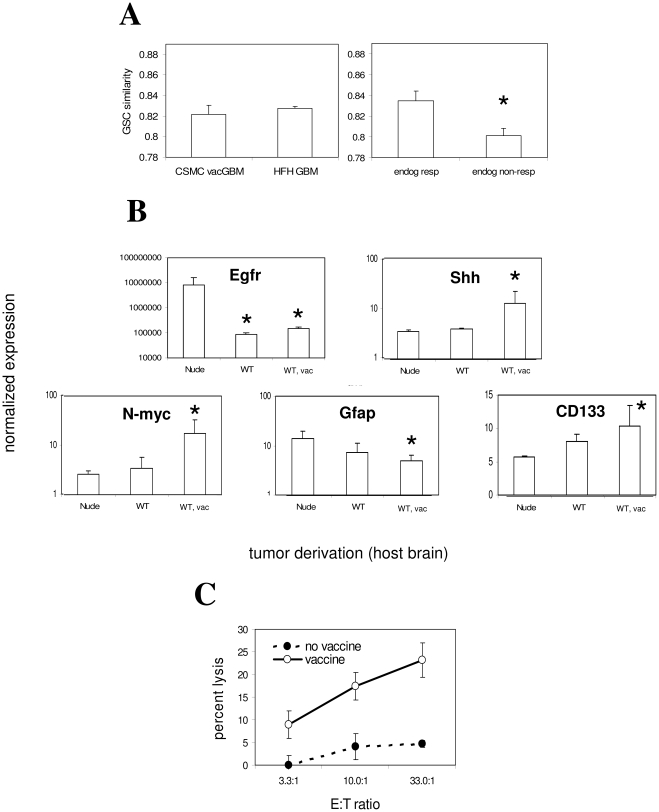
Regulation of stem-like gene expression in proportion to anti-tumor T cell activity. (A) CSC similarity (Pearson's coefficient for similarity to GSCs – GEO accession #GDS2728 - across all transcripts) from 200 Henry Ford Hospital GBM patients (GEO accession #GSE4536) and 6 CSMC GBM patients was assessed and found to be statistically identical, demonstrating absence of relevant bias in CSMC patients (left panel). Division of CSMC patients according to median pre-vaccine anti-tumor response levels as described [23] revealed significantly lower GSC similarity in low anti-tumor responders (0.84±0.01 vs. 0.81±0.01; *P*<0.05, one-tailed T-test; n = 3 per group), and this levels was also significantly lower than average of HFH patients (*P*<0.04; one-tailed T-test). (B) Quantitative PCR was performed using primers to the indicated genes, and products quantified by SybrGreen on an iCycler system (BioRad, Hercules, CA). RNA was derived from 3 independent low-passage sublines per strain (3–5 passages after brain recovery). Asterisks denote significant difference in GAPDH-normalized expression of each of the indicated genes in WT or WT, vac-recovered relative to nude-recovered GL26 cells by ANOVA. Gli1 expression was also marginally but significantly increased in GL26B6V relative to GL26nu (not shown; 18.9% relative to GAPDH; *P*<0.002; two-tailed T-test). All reactions were performed in triplicate for each individual tumor subline (3 sublines per host type). (C) CTL responses of immunocompetent and vaccinated GL26-bearing mice. CTL activity of splenocytes from glioma hosts. CTL activity of *ex vivo*-stimulated splenocytes (7 days) from non-vaccinated and vaccinated symptomatic, intracranial GL26-bearing mice against cultured GL26 cells was assessed by tetrazolium assay, and plotted as GL26 lysis–spontaneous (effector + responder)lysis/total lysis. Raw values at higher E:T ratios were significantly higher those of the lowest E:T ratio in non-vaccinated controls (*P*<0.02 for 10:1, *P*<0.003 for 33:1 E:T ratios, respectively; ANOVA).

It was not possible to directly determine how similarity to post-vaccine microarray profiles was acquired in GBM samples from non-vaccinated patients treated at other institutions. Nevertheless, we observed that endogenous anti-tumor IFN-γ responses [23] were directly correlated with GSC gene expression in our patients' samples prior to vaccination (Fig. 2A, Fig. S2B). This is consistent with the possibility that endogenous immune responses enhance stem-like gene expression. Together with the observation that GBM similarity to GSCs increases upon vaccination, this suggests that endogenous and/or vaccine-induced T cell activity are sufficient to enhance stem-like gene expression in gliomas.

Since non-vaccine therapies can increase stem-like properties in GBM [14], [15], we addressed whether non-specific post-treatment affects might account for increased stemness after vaccination. We therefore asked whether GSC gene expression was distinctly related to vaccine-associated or standard therapy-associated alterations in GBM gene expression. This was accomplished by plotting similarity to the GSC microarray profile (Pearson's coefficients across all transcripts) versus similarity to either post-vaccine or post-standard therapy microarray profiles (Pearson's coefficients across all transcripts) within 200 independent GBM samples (Fig. 1B), and generating exponential correlation coefficients as a measure of their inter-relatedness. This analysis aimed to discern whether GSC similarity was more related to vaccine-induced or standard therapy-induced expression profiles globally, but is not able to discern further details of what specific genes are involved in any relationships so identified. GSC similarity correlated significantly with both post-vaccine and post-standard therapy similarity. This suggested that stem-like gene expression is related to both vaccine and standard therapies, although perhaps more significantly to vaccine therapy (Figure 1B). To examine the basis of the potentially stronger relationship between stem-like and post-vaccine gene expression, we divided samples further into weakly and more strongly stem-like subgroups. This subdivision revealed that weak similarity to the CSC profile correlated equally with standard therapy- or vaccine-influenced gene expression, but higher CSC similarity correlated exclusively with the post-vaccine profile (Figure 1B). This is consistent with the possibility that a relatively low level of stem-like gene expression may be conferred onto GBM by either standard or vaccine therapy, but that vaccination confers a higher level of stem-like gene expression to these tumors.

To identify oncogenic pathways potentially involved in T cell-influenced gene expression, we performed Ingenuity Pathway Analysis (IPA) on vaccine-treated GBM and GL26. IPA is a software tool that extracts and categorizes multiplexed information such as gene expression microarray data into groups based on known functional involvement of individual components (i.e., individual genes) as documented in scientific literature. Such analysis quantifies pathway involvement by calculating the proportion of genes within a number of defined pathways that are altered by a given treatment or manipulation. IPA of transcripts significantly altered after vaccination in GBM patients and/or in GL26 recovered from DC vaccine-treated mice revealed that 7 of the top 11 developmental/oncogenic pathways were up-regulated in common between human GBM and mouse GL26 (Fig. S3). In this context, Sonic Hedgehog (Shh), a pathway implicated in glioma CSCs [8], [14], [30], [31], [32], [33], was particularly affected by vaccination (Fig. S3). Similarly, 6 of the top 11 pathways were down-regulated in common between GBM and GL26 after vaccination, with particular prominence of Egf and Erk/MapK pathway components (Fig. S3). These data suggest that vaccination consistently up-regulates developmental/oncogenic pathways such as Shh, while distinct oncogenic pathways such as Egfr may be consistently down-regulated.

Shh, CD133 and N-myc up-regulation characterize a highly oncogenic GSC population [1], [2], [4], [8], [14], [15], [34]. We utilized GL26nu, GL26B6, and GL26B6V sublines to assess the influence of absent, weak, and strong anti-tumor T cell activity, respectively, on these genes, and verified up-regulation of Shh, N-myc, and CD133 in GL26B6V relative to GL26nu by quantitative PCR (qPCR; Fig. 2B and legend). In contrast, Egfr and Gfap expression were significantly decreased in GL26B6V relative to GL26nu. Moreover, GL26B6 exhibited more modest regulation of these same genes, in the same direction as GL26B6V (Fig. 2B). Since non-vaccinated wild-type mice exhibited weak endogenous anti-tumor T cell activity by CTL assay, and such activity was substantially increased upon vaccination (Fig. 2C), this suggested that stem-like gene expression in gliomas is modulated in direct proportion to anti-tumor CTL activity levels.

**Figure 3:**
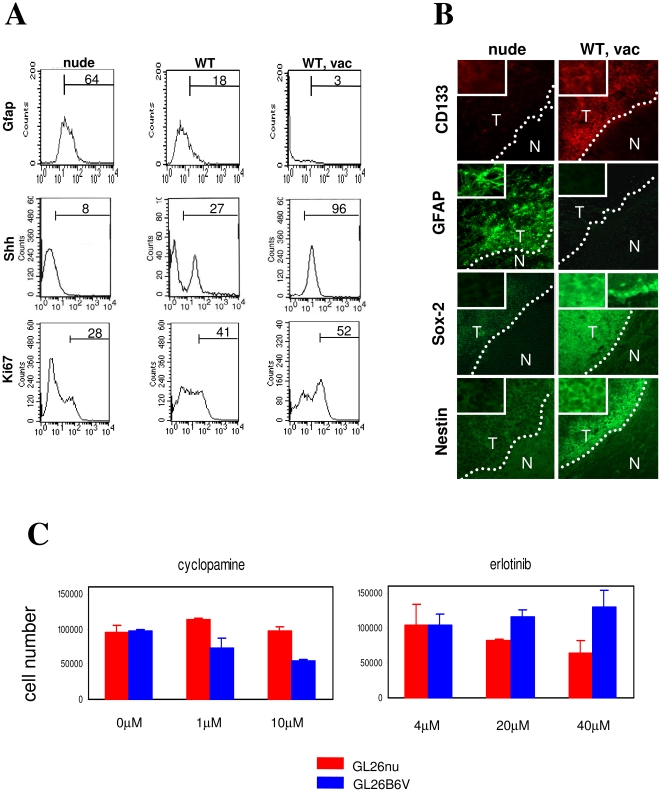
Stem-like protein expression and function modulated by T cell activity. (A) Flow cytometric staining and analysis was performed on GL26 tumor section from brains of nude (GL26nu), C57BL6J (WT; not shown), and C57BL/6J mice vaccinated with 2×10^6^ GL26 lysate-pulsed DC2.4 cells 3 and 7 days post-tumor implantation (“WT, vac”). Percentage of positive cells appears above gating bar. Markers shown had significantly different (*P*<0.05) positive cell percentages in 3 independent samples of GL26nu and GL26B6V cells by ANOVA and/or one-tailed T-test. (B) Immunofluorescence staining was performed on GL26 tumor section from brains of nude, and C57BL/6J mice vaccinated with 2×10^6^ GL26 lysate-pulsed DC2.4 cells 3 and 7 days post-tumor implantation (“WT, vac”). Results are representative of ≥ samples/group, with the exception of CD133, which revealed strongly positive tumor staining in 2 of 4 brains. WT brains generally exhibited staining similar to nude, or intermediate staining between that of nude and WT, vac (not shown). Marker expression of WT, vac (Prominin^+^, GFAP^-^, Sox-2^+^, Nestin^+^) is characteristic of cancer stem cells. However, Sox-2 expression was not isolated to nuclei in WT, vac GL26 (right panel, top left inset), though Sox-2 staining of contralateral ventricular cells in the same brain was (right panel, top right inset). (C) Cell numbers in absence or presence of the indicated concentrations of Erlotinib (Tarceva) or Cyclopamine were determined for low-passage (≤5) GL26nu and GL26B6V by Coulter counter. Differences between GL26nu and GL26B6V for either drug, and between erlotinib and cyclopamine within the same recovered tumor lines were significant (*P*<0.03 by one-tailed T-Test and ANOVA), at concentrations above 4 uM. Distinct glioma lines exhibited opposite patterns of drug sensitivity: GL26nu cells were sensitive to erlotinib but not to cyclopamine, and GL26B6V cells were sensitive to cyclopamine but not to erlotinib, consistent with their reciprocal expression of EGFR and SHH target genes.

Flow cytometry verified increased expression of Shh protein, as well as of Ki67, a proliferative marker associated with CSC malignancy [35], as well as decreased expression of Gfap protein in GL26B6V relative to GL26nu. Incremental modulation of these proteins was also observed in GL26B6 (Fig. 3A). Immunohistochemical analysis verified that GL26 brain tumors *in situ* were CD133^+^ and Gfap^−^ in vaccinated WT but not in nude mice (Fig. 3B). Nestin and Sox-2, additional stem cell proteins, were also up-regulated on *in situ* tumors in vaccinated WT mice (Fig. 3B). Expression of Sox-2, however, was atypical in that it was not confined to the nucleus (Fig 3B, Sox-2 right panel, top left inset), although staining of contralateral ventricular cells with the same antibody revealed predominantly nuclear Sox-2 expression (Fig 3B, Sox-2 right panel, top right inset). Thus, vaccine-exposed GL26 may retain Sox-2 in the cytoplasm and/or fail to efficiently transport it to the nucleus, which may limit Sox-2 transcriptional activity in these cells.

### T cell-exposed GL26 gliomas exhibit enhanced cyclopamine sensitivity, chemoresistance, and tumorigenesis

The above data suggest that exposure to anti-tumor T cell activity, induced endogenously or through DC vaccination, renders gene expression more stem-like in gliomas. To determine whether exposure to anti-tumor T cell activity mediates stem-like functional properties, we took advantage of recent evidence suggesting that stem-like glioma cells are depleted by the Shh antagonist, cyclopamine [33]. We thus treated GL26nu and GL26B6V cells (unexposed or exposed to strong anti-tumor T cell activity, respectively) with cyclopamine, and with Tarceva (Erlotinib), an inhibitor of Egfr signaling used here as a control. Cultured GL26B6V cells grew with Tarceva treatment but were depleted by cyclopamine treatment (Fig. 3C), suggesting relative resistance to Egfr and sensitivity to Shh inhibition. In contrast, GL26nu were unaffected by cyclopamine but were depleted by Tarceva treatment (Fig. 3C), suggesting relative resistance to Shh and sensitivity to Egfr inhibition. These data suggest that exposure to anti-tumor T cell activity renders glioma growth increasingly sensitive to Shh inhibition, and less sensitive to Egfr inhibition.

**Figure 4:**
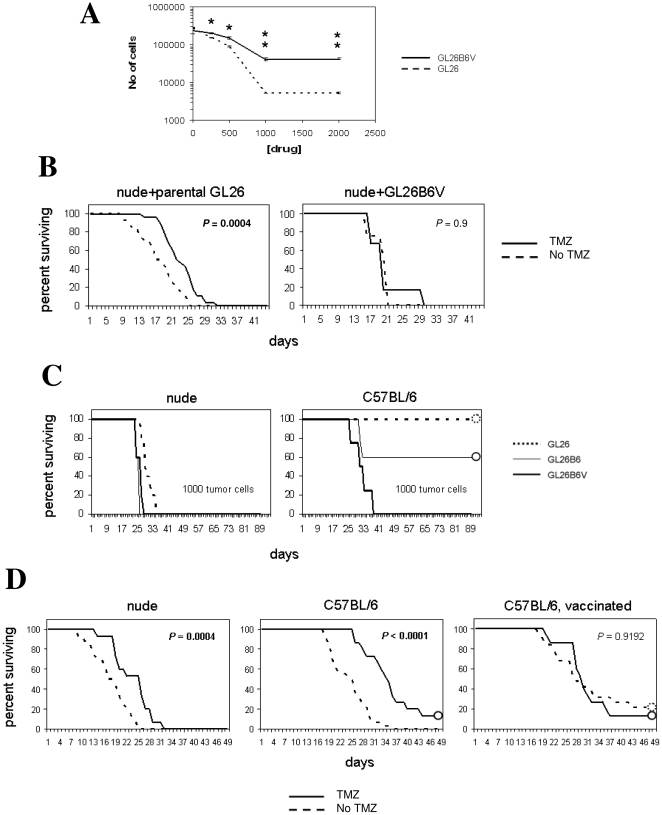
*In Vitro* and *in vivo* drug resistance of vaccine treated vs. non-treated tumor cells. A) Cell numbers ± indicated concentrations of temozolamide were determined for low-passage (≤5) GL26nu and GL26B6V with a Coulter counter. Cell numbers were significantly different (*P*>0.7; one-tailed T-test) between GL26nu and GL26B6V at all drug concentrations, and trends were significantly different among 3 independent sublines of these tumors. (B) Female nude mice (Harlan, Inc.) were injected intracranially with 50,000 GL26 tumor cells, or 50,000 GL26B6V tumor cells, treated by i.v. injection 7, 8, & 9 days post-tumor implantation with 10 mg/kg temozolamide (TMZ) in 1% DMSO, and days to survival assessed by log-rank. GL26-implanted mice survived significantly longer (*P*<0.003) with temozolamide treatment, while GL26B6V-implanted mice did not (*P*>0.7). (C) Female wild-type C57BL/6J (Jackson Labs; right panel) or nude mice (Harlan, Inc.; left panel) as indicated were injected intracranially with indicated doses of GL26 (n = 5), GL26B6 (n = 5), or GL26B6V (n = 5) tumor cells, and days to survival assessed by log-rank. GL26-implanted survived significantly longer (*P*<0.0001; Mantel-Cox log-rank) than GL26B6V-implanted wild-type mice at doses up to 50,000 cells (not shown), but only just achieved significantly longer survival in nude mice at doses of 25 cells (*P* = 0.049; Mantel-Cox log-rank, not shown). (D) Survival between GL26-implanted nude (n = 28), untreated C57BL/6J (n = 32), and C57BL/6J mice vaccinated with 2×10^6^ GL26 lysate-pulsed DC2.4 cells 3 and 7 days post-tumor implantation (n = 19) was assessed with and without subsequent temozolamide (TMZ) treatment as above, by Mantel-Cox log-rank statistics. DC-vaccinated hosts survived significantly longer relative to untreated wild-type and nude mice implanted simultaneously with 50,000 original GL26 tumor cells (*P*<0.003).

Previous reports also indicate that GSCs are chemoresistant. Viability of GL26nu and GL26B6V was examined with and without temozolamide (TMZ), a standard GBM chemotherapeutic to which classical GSCs are resistant, to assess whether exposure to anti-tumor T cells activity induces stem-like drug resistance. TMZ depleted both GL26nu and GL26B6V in a dose-dependent manner *in vitro*, but GL26B6V was far less sensitive than GL26nu (Fig. 4A). BL26B6 exhibited intermediate TMZ depletion (Fig. S4A). Together, these results suggest that exposure to anti-tumor T cell activity proportionally increases glioma chemoresistance *in vitro*. In addition, TMZ extended survival of nude mice implanted with parental GL26, but not of those implanted with GL26B6V (Fig. 4B), suggesting that exposure to strong anti-tumor T cell activity increases glioma chemoresistance *in vivo*. Thus, vaccine-exposed GL26, like classical glioma stem cells, appear sensitive to cyclopamine as well as chemoresistant.

We next examined tumorigenicity of vaccine-exposed GL26, as enhanced tumorigenicity is a consistently reported property of GSCs. Limiting doses of GL26B6V cells were rapidly lethal in wild-type mice, whereas the same dose of GL26B6 was less tumorigenic, and parental GL26 were non-tumorigenic (Fig. 4C). Thus, prior exposure of gliomas to increasing levels of T cell activity proportionally enhanced their tumorigenicity upon re-implantation into immunocompetent hosts. By contrast, implantation of limiting doses of parental GL26, GL26B6, and GL26B6V did not reveal distinct tumorigenicity in T cell-deficient (nude) hosts (Fig. 4C; Fig. S4B), as expected of classical GSCs [1], [2], [8], [33], [36], [37]. These findings raised the possibility that vaccine-enriched glioma cells might be somewhat distinct from classical GSCs enriched by chemotherapy or neurosphere culture. Underscoring this possibility, DC vaccination enhanced the survival of GL26 hosts to a greater extent than TMZ (Fig. 4D),

To help clarify the relationship between vaccine-enriched glioma cells and classical GSCs, we sought to determine whether treatment with DC vaccine or with TMZ, a chemotherapeutic that enriches classical GSCs (Fig. 4D) [15], enriched the same stem-like glioma population. We reasoned that the 2 therapies should synergize only if they eliminated or otherwise acted on distinct tumor subpopulations. GL26 was therefore implanted simultaneously into WT and nude mice, followed by TMZ treatment and/or DC-vaccination (WT only). TMZ significantly enhanced survival in both nude and WT hosts, with maximal enhancement in unvaccinated WT, but did not synergize with vaccination (Fig. 4D). This pattern of synergy is consistent with a scenario in which vaccine-induced T cell activity acts on the same tumor subpopulation targeted by TMZ chemotherapy, but in which endogenous T cell activity acts on tumor cells that survive or are unchanged by chemotherapy.

**Figure 5:**
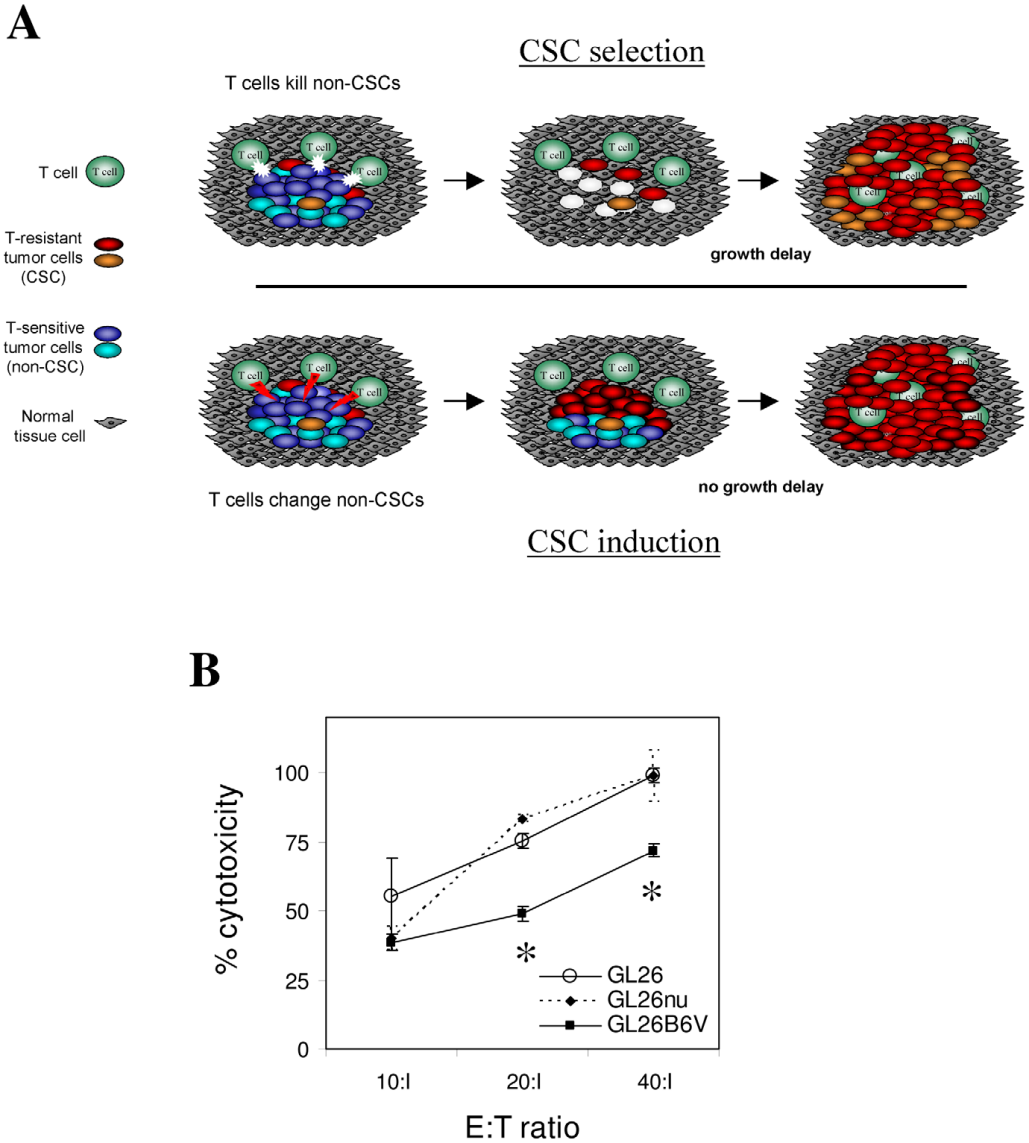
Selection vs. induction of stem-like properties in gliomas. (A) Expected appearance of gliomas under alternative mechanisms of selection of pre-existing glioma stem cells (top panel) vs. induction of stem-like genetic program (bottom panel) via vaccination. The present data best support the occurrence of immune-mediated glioma CSC selection with or without prior immune induction of these cells. (B) Vaccine-exposed (GL26B6V) and parental GL26 were co-incubated with indicated ratios of HTB-156.7.7 (an H-2K^b^-reactive T hybridoma [39]), and assessed for killing. A representative of 3 independent assays is shown. GL26B6V exhibited relative resistance to killing, consistent with a selection mechanism for T cell-mediated enrichment of stem-like gliomas. Asterisks denote significantly reduced specific lysis in triplicate wells (*P*>0.05 by single-sided T-test).

The above survival study also indicated that host survival is prolonged by DC vaccination, despite its promotion of tumor cells with enhanced tumorigenicity in immunocompetent hosts (Fig. 4C). This may occur because vaccination eliminates a larger or more malignant non-stem tumor subpopulation than the stem-like tumor cells it spares. If true, this would implicate selection rather than induction as a mechanism for T cell-mediated enrichment of GSCs, as outlined in Fig. 5A. A prerequisite for such immune-mediated selection is relative resistance of stem-like tumor cells to T cell killing. In this context, killing of GL26B6V by the allo-reactive T cell hybridoma, HTB-157.7 [38] [39] [40] [41] was partially diminished at high effector:target (E:T) ratios relative to both parental GL26 and to GL26nu (Fig. 5B). Although further studies are necessary to determine whether such CTL resistance is sufficient to account for T cell-mediated enrichment of GL26B6V, its relative resistance to T cell killing is consistent with immune-mediated selection.

### Genetic heterogeneity in T cell-exposed GBM and GL26 gliomas

**Figure 6:**
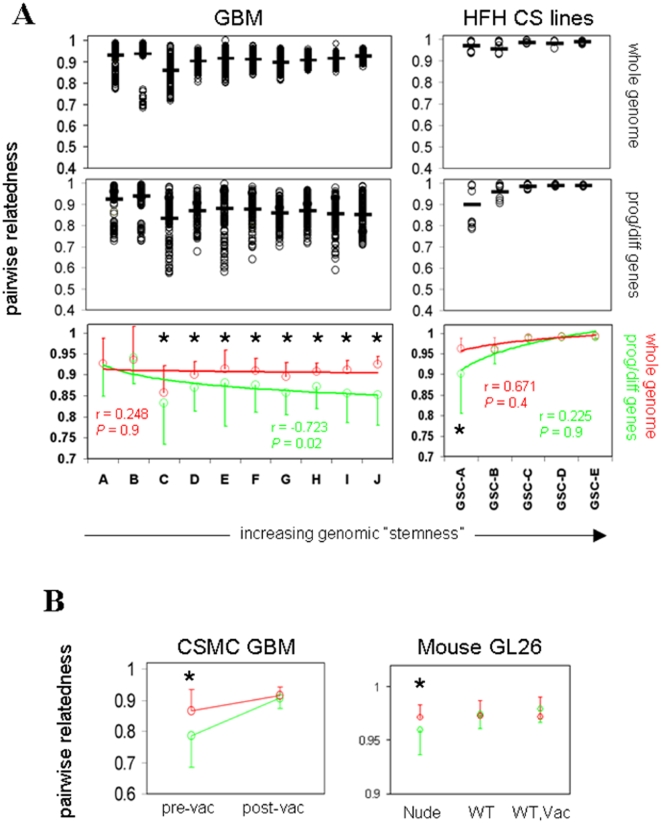
Genetic diversity and stem-like properties in GBM. (A) Henry Ford Hospital GBM (“GBM”, total n = 200), and cultured GBM lines grown in stem cell media (“HFH CS lines”, total n = 23) as indicated (GEO accession #GSE4536 for both), were arranged in groups with increasing global CSC similarity as in Fig. 1B (n = 20/group for GBM, groups A-J; n = 5 for 4 groups, and n = 3 for the most stem-like group for GBM progenitors; groups GSC-A through -E), each with progressively increased CSC similarity, and intra-group pair-wise relatedness assessed by Pearson's correlation matrix across all transcripts, and within genes involved in differentiation/progenitor cell function (publically posted by Superarray, Inc.) as indicated. Range and average (horizontal bar) of individual coefficients are plotted in uncolored panels, with compiled averages ± SEM for all transcripts and differentiation/progenitor genes combined in colored panels. Significant differences in intra-group similarity between the two gene sets are denoted by asterisks (*P*<0.01; ANOVA). (B) Pre- and post-vaccine CSMC GBM (left panel; n = 6 matched pairs), and mouse GL26 microarray samples recovered from nude, syngeneic C57BL/6 (“WT”), or DC-vaccinated C57BL/6 (“Vac”; right panel; n≥4/group, as described in Fig. 1A), were grouped and assessed for intra-group pair-wise relatedness by generating Pearson's correlation matrices across all transcripts, or for differentiation/progenitor cell genes as above. Compiled averages ± SEM for all transcripts (red) and differentiation/progenitor genes (green) were plotted, with significant differences between these two parameters denoted by asterisks (*P*<0.01; ANOVA).

It has been suggested that, by promoting tumor cell differentiation, GSCs may enhance genetic heterogeneity within GBM (1). If true, increasingly stem-like tumors should exhibit increased heterogeneity, particularly within genes involved in progenitor cell function and/or differentiation. To test this, we stratified 200 GBM samples into 10 subgroups based on increasing GSC similarity. We then assessed pair-wise relatedness of samples within each subgroup across all transcripts (Pearson's correlation coefficients for every possible sample pair within each stratified group, considering all probesets) as well as within genes expressed in cell progenitors or during differentiation (progenitor/differentiation genes; Pearson's correlation coefficients for every possible sample pair within each stratified group, considering only thos probesets known to be expressed in cell progenitors or during differentiation). The aim of this analysis was to determine the degree of gene expression heterogeneity potentially related to differentiation, between GBMs from different patients as a function of their stemness.(Fig. 6A). Intriguingly, increasing GSC similarity did not consistently increase intra-group similarity across all transcripts, and was even associated with progressively decreased intra-group similarity (increased heterogeneity) within progenitor/differentiation genes (Fig. 6A; group GSC-A). Thus, heterogeneous expression was selectively increased within genes potentially related to differentiation, and in direct relation to stemness. This helps validate the notion that possession of a stem-like tumor genetic profile enhances GBM genetic heterogeneity *in situ*.

In contrast, 23 GBM cell lines grown in stem cell media and stratified into 5 subgroups based on increasing GSC similarity exhibited uniformly high intra-group similarity (i.e., relative homogeneity) across all transcripts and within progenitor/differentiation genes (Fig. 6A). These data demonstrate that stem-like GBM lines exhibit evidence of homogenized gene expression, whereas stem-like GBM tumor tissue exhibits heterogeneity within progenitor/differentiation genes. Such heterogeneity could be ascribed to contaminating non-stem subpopulations and/or transitional cell states accompanying stem-like GBM *in situ* that are subsequently lost in culture. Alternatively, it could be due to enrichment of distinct categories of stem-like tumor cells by different environmental constraints.

Because stem-like gene expression in GBM was related to endogenous rather than vaccine-induced anti-tumor immunity, it was unclear how vaccine exposure would affect GBM heterogeneity within progenitor/differentiation genes. We thus examined heterogeneity among pre- and post-treatment (vaccine or standard therapy) GBM pairs from the same patients. Surprisingly, the relatively high heterogeneity within progenitor/differentiation genes observed in non- or pre-vaccinated GBM was eliminated in tumors acquired from the same patients after DC vaccination (Fig. 6B). Thus, regardless of the specific etiology of this heterogeneity or its role in GBM phenotypic variation, GBMs acquired more homogeneous gene expression after exposure to strong anti-tumor T cell activity.

Whereas these results suggest that strong anti-tumor immune activity has the ability to homogenize expression of genes potentially involved in progenitor cell function and/or differentiation, it was still unclear whether the absence of T cell activity promote heterogeneity within these genes. In this context, the pre-vaccine heterogeneity observed in GBM patients could have originated from distinct genetic origins of tumors in different patients, rather than from the absence of T cell activity per se. To further clarify the potential impact of anti-tumor T cell activity on glioma genetic heterogeneity, and to formally address whether the lack of anti-tumor T cell activity can enhance heterogeneous gene expression in glioma, we assessed microarray profile similarity among biological replicates of GL26nu, GL26B6, and GL26B6V (all dervived from the same parental tumor). Remarkably, relatively high heterogeneity within progenitor/differentiation genes was observed only in GL26nu (Fig. 6B). In contrast, GL26B6 and GL26B6V exhibited more homogeneous gene expression within progenitor/differentiation genes (Fig. 6B). Together, these findings suggest that strong anti-tumor T cell activity is sufficient to render gliomas more stem-like and to homogenize progenitor/differentiation gene expression. Similarly, the absence of anti-tumor T cell activity can enhance heterogeneous expression within the same set of genes in gliomas.

## Discussion

Microarray analyses (PCA and IPA) on all transcripts, vaccine-altered, and immune-related genes, suggested that *in vivo* vaccine treatment rendered GBM gene expression more similar to that of classical GSCs (Fig. 1, Fig. S2). We considered the possibility that these results may be dependent on the use of a tumor lysate-pulsed DC vaccine strategy to elicit anti-tumor CTL activity. In this context, however, endogenous anti-tumor T cell activity measured prior to vaccination [23], correlated directly with GBM stemness (Fig. S2B), suggesting that increased T cell activity in general (endogenous or DC vaccine-induced), correlated with a stem-like gene expression profile in these patients. Nevertheless, it is recognized that specific targeting of GSC antigens during T cell induction may yield distinct findings. For example, others have previously shown that DCs loaded with stem-like, as opposed to parental, glioma cells are better able to elicit an effective immune response against either parental or stem-like gliomas in mice [42] [43]. Such treatment would not necessarily be expected to increase stem-like properties in surviving tumors, particularly if T cell activity enriches glioma stemness solely through selective killing of targeted tumor cells. This raises the possibility that our own vaccination strategy enriched GSCs because it failed to include T cell epitopes of GSCs. However, we and others have observed that anti-tumor T cells in DC-vaccinated human and mouse glioma hosts predominantly recognize Trp-2, or dopachrome tautamerase (DCT; [44] [27]), which has been shown to enhancee stem-like properties in neural progenitors [45]. The molecular mechansism whereby T cell activity enhances GSC properties in GBM is therefore unclear, and requires further study to clarify whether it proceeds via selective destruction of tumor cells and/or by independent means.

Stem-like properties were assessed in the transplantable murine glioma, GL26 to determine if T cell activity directly caused an increase in glioma stemness. GL26 exhibited more stem-like gene and protein expression (Figs. 1A; 2B; 3A, B; Fig. S2A), as well as GSC functions including cyclopamine sensitivity (Fig. 3C), chemoresistance (Fig. 4A, B), and enhanced tumorigenesis in wild-type hosts (Fig. 4C), after exposure to anti-tumor CTL activity derived from either endogenous or vaccine-induced T cell responses. Specifically, GSC-like gene expression, protein expression, chemoresistance, and tumorigenesis and functional properties were incrementally but consistently increased in GL26 gliomas exposed to low but measurable endogenous anti-tumor CTL activity (GL26B6), and such properties were more dramatically increased in GL26 exposed to higher DC vaccine-induced anti-tumor CTL (GL26B6V; Fig. 2B; 3A; 4C; Fig. S4A, B). Together, these data indicate a strong correlation between anti-tumor T cell activity and stem-like properties in clinical glioma specimens. In addition, the observation that T cell presence caused stem-like properties to increase in GL26 in direct proportion to the level of anti-tumor CTL activity against this glioma, suggests that stem-like properties may be directly enhanced by anti-tumor T cell activity. As such, these data represent the first evidence that tumor stemness may be regulated by inducible physiological processes generally, and anti-tumor CTL activity in particular. Nevertheless, further investigation is required to elucidate the precise nature and involvement of anti-tumor T cell activity in enhanced stem-like properties in gliomas.

Shh and EGFR signaling pathways, each of which can mediate brain tumor generation and/or malignancy, were identified as the most prominently up- and down- regulated oncogenic pathways, respectively, common to GBM and GL26 exposed to T cells *in vivo*. (Fig. S3). Furthermore, sensitivity to cyclopamine a Shh signaling antagonist, (Fig. 3C), *in vitro* and *in vivo* chemoresistance, and resistance to CTL killing were also enhanced in vaccine-exposed GL26 (GL26B6V; Fig. 4A, B, Fig. 5B). These data suggest that vaccine-exposed gliomas may be generally resistant to chemical and immune cytotoxicity, but maintain sensitivity to anti-stem agents including cyclopamine. It is therefore tempting to speculate that anti-stem agents might be particularly useful in combination with vaccination to treat malignant gliomas. It is equally intriguing to speculate that a switch in dependency from EGFR-mediated to Shh-mediated growth might critically impact the acquisition of stem-like functional properties, and/or tumor immune resistance, and the role of each of these pathways in such phenomena are subjects of further studies.

In this study, we observed that greater stemness, as defined by microarray similarity, was inversely associated with brain tumor malignancy (Fig. S1C). Careful comparison of published literature indicates that somewhat comparable patterns have been previously observed by others [1], [2]. Specifically, limiting dilution analysis of multiple medulloblastomas, and GBMs by the same authors [1], [2] reveal consistently lower propensity of GBM relative to medulloblastomas to form neurospheres enriched for tumor initiating tumor cells, although low grade astrocytomas also generated few neurospheres [1], [2]. In contrast, we and others observed a loose correlation between CD133 expression and glioma stemness, yet significantly higher CD133 expression in high-grade relative to low-grade gliomas (Fig. S1C)[1], [2]. These distinct tumors all grow in different ways, within distinct parts of the brain, which may impact malignancy. Moreover, the cell of origin for these tumors is hypothesized to be distinct, which may be a major factor impacting the frequency of CSCs in each tumor type. Thus, the relationship of CD133 expression and other metrics to stem cell properties of brain tumors may be complex, and therefore requires further clarification.

**Figure 7:**
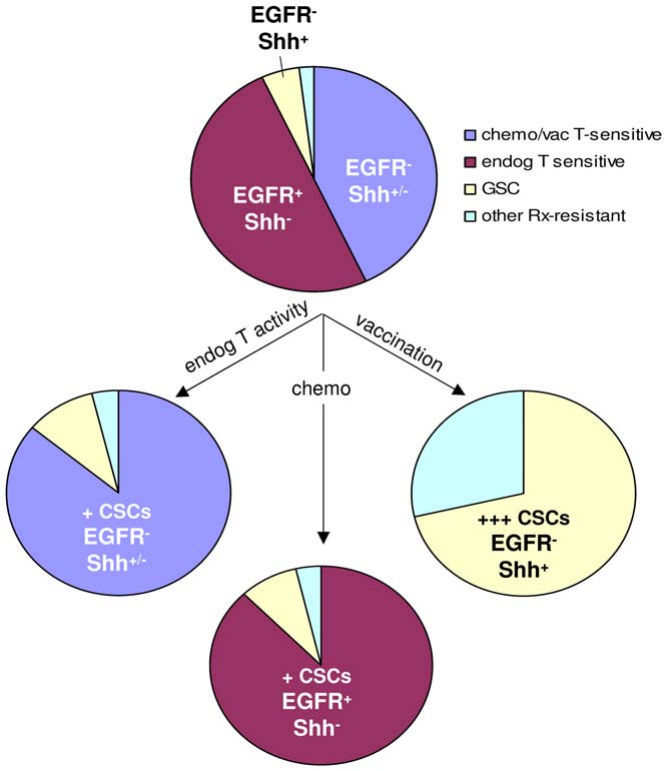
Model for vaccine-induced stem-like properties in gliomas. Model accounting for qualitatively and quantitatively distinct enrichment of glioma CSCs by endogenous T cells, chemotherapy and vaccination. The model takes distinct synergy of endogenous and vaccine-mediated T cell activity with chemotherapy, and therapy-mediated marker dynamics into account. In the absence of any T cells, substantial numbers of Egfr^+^ tumor cells are expected to persist, whereas T cells down-regulate Egfr and proportionally up-regulate Shh expression.

We observed a superior correlation between vaccine-induced, as opposed to standard therapy-induced, and GSC gene expression (Fig. 1B). In this context, vaccination, like chemotherapy, appeared to enhance stem-like properties in gliomas, yet significantly benefited murine, and perhaps human, host survival (Fig. 4D)[16], [17], [23]. Together, these observations suggest that enrichment of stem-like gliomas might be disassociated at least under certain physiological conditions from greater tumor malignancy, a notion that is also consistent with GSCs exacerbating malignancy within discrete GBM subcategories [19]. Such disassociation was evident in vaccine-exposed GL26 (GL26B6V) growing in nude hosts, which was no more lethal than their counterparts (GL26, GL26nu; Fig. 4C). In this respect, vaccine-exposed gliomas appeared somewhat distinct from classical GSCs defined by CD133 expression and/or neurosphere selection. Since classical GSCs are also enriched by treatments such as chemotherapy, we would expect vaccination and chemotherapy to synergize in the event they eliminated distinct GSC subpopulations. Nevertheless, the lack of synergy between vaccination and chemotherapy in mice is consistent with chemotherapy sparing the same stem-like tumor subset as vaccination (Fig. 4D). Together with the apparent synergy between endogenous T cell activity and chemotherapy, this supports a model in which endogenous anti-tumor T cell activity and standard chemotherapy, may each eliminate distinct individual non-stem tumor subpopulations, whereas vaccine-induced T cell activity eliminates both. In this manner, both vaccination and chemotherapy could enrich stem-like tumor cells, but vaccination would do so more efficiently. Thus, chemotherapy-exposed and vaccine-exposed gliomas may harbor similar stem-like components, but each may also be accompanied by distinct non-stem tumor subpopulations, as illustrated in Fig. 7.

Our findings are most consistent with a selective rather than inductive model of stem-like tumor enrichment by T cell activity, which specifically predicts a delay in tumor growth afforded by DC vaccination (Fig. 5A), as well as selective CTL resistance of stem-like glioma cells. Both properties were observed using the GL26 system (Fig. 4D, 5B). Nevertheless, further studies are required to fully test whether GSCs are enriched by DC vaccination solely via selection, as it is unclear whether differential killing of GL26B6V at the relatively high E:T ratios presented here is sufficient to entirely account for vaccine-mediated promotion of stem-like properties in GL26. Differential susceptibility to cytolytic therapeutics including radiation and chemotherapy also enrich classical GSCs [14], [15], but the relationship between these and vaccine-elicited gliomas requires further clarification. An additional possibility is that decreased immunogenicity of stem-like GL26 cells contribute to their enrichment after DC vaccination. In this context, we have observed no quantitative or qualitative difference in dominant antigen-directed T cell responses mediated by re-implantation GL26B6V relative to parental GL26 into wild-type mouse brains (59±5% vs 53±5% Trp-2-reactive intracranial CD8^+^ T cells, respectively; n = 7 mice each for GL26 and GL26B6V; *P* = 0.23, single-side T-test; not shown). Thus, we believe CTL resistance may play a more prominent role in enrichment of stem-like GL26 than reduced immunogenicity.

Despite globally homogeneous gene expression in classical GSCs, significantly higher heterogeneity within progenitor/differentiation genes was evident in more stem-like GBM from external sources (Fig. 6A). This is consistent with the notion that GSCs may contribute to GBM heterogeneity by promoting heterogeneous expression within genes involved in progenitor cell function and/or differentiation. Similarly compartmentalized heterogeneity was also evident in our patients' GBM tissue prior to DC vaccination, but was significantly decreased in GBM tissue acquired after DC vaccination from the same patients (Fig. 6B). The basis for why stem-like GBM tissue should be associated with heterogeneous expression in progenitor/differentiation genes, whereas DC vaccination that apparently increases stemness in these tumors is associated with decreased heterogeneity is unclear, but could reflect the persistence of distinct contaminating tumor subpopulations co-enriched with GSCs *in situ*. Alternatively, the discrepancy could reflect that classical GSCs, being enhanced by relatively weak endogenous anti-tumor T cell activity, may differ qualitatively from GSCs enhanced by strong vaccine-mediated T cell activity. Further studies are needed to critically test these possibilities. More practically, however, compartmentalized heterogeneity within progenitor/differentiation genes could conceivably allow tumors to evade targeted or conventional treatments via outgrowth of resistant tumor variants, whereas homogenized gene expression could potentially enhance susceptibility to such therapies. In this context, the findings of progenitor/differentiation gene homogenization after vaccination, coupled with more heterogeneous expression of these genes under conditions of T cell deficiency in GL26, are particularly intriguing in that they point to a previously undocumented ability of immune activity to constrain tumor genetic variation. Although the relationship of heterogeneous expression within the progenitor/differentiation gene set examined to phenotypic heterogeneity in gliomas has yet to be established, reduced genetic variation in general could conceivably impact adaptive tumor responses and thereby stabilize therapeutic efficacy in GBM.

Together these findings provide the first evidence that tumor stemness can be regulated by a targetable physiological process, namely anti-tumor T cell activity. Moreover, they highlight that immune resistance, like chemoresistance, may be accompanied by greater reliance on stem cell signaling pathways. Moreover, they provide evidence that tumor malignancy may not be directly associated with stem-like tumor components in all cases. Further study is required to establish the precise relationship between vaccine-elicited gliomas and classical GSCs, and between non-stem and stem-like tumor components, but manipulation of physiological processes inducing glioma stemness promises continued clarification of GSC origins, treatment susceptibility, and their role in tumor malignancy.

## Supporting Information

Figure S1 Validation of GSC similarity to distinguish CSCs from non-fractionated GBM. (A) GSC similarity (Pearson's coefficient for similarity to GSCs across all transcripts), and CD133 expression, were determined for GBM from HFH (n = 200) and high-grade gliomas from UCLA (n = 45; GEO accession #GSE4412), plotted against each other for each individual sample, trendlines generated, and r and P values determined as depicted. CSC similarity correlated significantly with CD133 expression within two separate databases. (B) Ability of CD133 expression, or GSC similarity, to distinguish non-fractionated GBM from CD133^−^ or CD133^+^ GSCs (29) (GEO accession #GDS2728) or from stem cell media-cultured GBM lines from 2 patients (37) (GEO accession #GSE4536); was determined (P<0.01 denoted by red asterisk). Unlike CD133 expression, only GSC similarity distinguished CD133^+^ or CD133 GSCs (from multiple sources) from surgical GBM samples (C) GSC similarity (Pearson's coefficient for similarity to GSCs - GEO accession #GDS2728 - across all transcripts), and CD133 expression, were determined for de novo GBM, secondary GBM, and grade 3 gliomas from UCLA (GEO accession #GSE4412) and each parameter assessed for inter-group differences by one-tailed T-test (P<0.01 denoted by red asterisk). CD133 expression has been shown to be highest in de novo GBM (29), and this was arguably the case in the samples we analyzed, but global GSC similarity was highest in grade 3 gliomas and secondary GBM (Fig. S1C). Global GSC similarity thus paralleled increased numbers of CSCs reported for lower-grade brain tumors (1, 2). These data validated that gene expression similarity distinguishes stem-like gliomas more faithfully than does CD133 expression.
(1.87 MB TIF)

Figure S2 Stem-like gene expression accompanies endogenous and vaccine-induced anti-tumor T cell activity in gliomas. (A) Principal Component Analyses focused on discrete gene lists were plotted in GenespringGX7.3, and group clusters circled, on the following: 59 GBMs from UCLA database (“UCLA GBM”), 12 GBMs from 6 patients collected before and after DC vaccination (“vaccinated GBM”); 10 GBMs from 5 patients collected before and after standard radiation and/or chemotherapy (“control GBM”) (red); CD133 and CD133^+^ CSCs from 6 University of Regensberg GBM patients (29) (“UR GSC”) (green); stem cell media-cultured lines from 2 Henry Ford Hospital GBM patients (“HFH CS lines”); murine GL26 glioma samples recovered and cultured <8 passages from brains of 5 nude (GL26nu), 4 C57BL/6J (GL26B6) and 4 C57BL/6J mice vaccinated with 107 tumor lysate-pulsed DC2.4 cells 3 and 7 d post-tumor implantation (GL26B6V). Post-vaccine GBM uniquely exhibited co-clustering (relatedness) with UCLA glioma progenitors within genes involved in immune modulation (left, middle panels). GL26B6V exhibited parallel clustering in the analogous mouse gene list (right panel). (B) CSC similarity (Pearson's coefficient for similarity to GSCs across all transcripts) of GBM patients for whom IFN-γ anti-tumor response data was known prior to vaccine therapy was plotted against pre-vaccine anti-tumor response levels as described (23) revealing a significant direct correlation between CSC similarity and response magnitude. (C) Matched microarray data from the 12 vaccinated GBM (Vaccine Rx), and the 10 control GBM (Standard Rx) were assessed for similarity to averaged expression values of UR GSCs (29) (“UR”), or to 20 HFH CS lines (“HFH”), and Pearson's coefficients across 54,674 transcripts plotted for each patient in line plots. Significantly increased stem-like gene expression (asterisks) was unique to post-vaccine samples (P<0.001; one-tailed T-test). See Fig. 1 legend for GEO accession numbers.
(1.79 MB TIF)

Figure S3 Ingenuity Pathways Analysis of vaccine-altered glioma genes. The involvement of vaccine-altered genes in 20 pathways containing known oncogenes is scored by a ratio of the number of vaccine-altered genes to the number of genes in each pathway. (A) Human GBM post-vaccine up-regulation. (B) Murine GL26 post-vaccine up-regulation. (C) Human GBM post-vaccine down-regulation. (D) Murine GL26 post-vaccine down-regulation. Up and down regulated genes were scored separately. Shown here are the 11 pathways with the highest ratios of changed genes to total genes among 20 pathways of interest. Signaling Pathways: Apoptosis, Death Receptor, EGF, ERK/MAPK, Estrogen Receptor, GF-1, NF-kappaB, Notch, p38 MAPK, PDGF, PI3K/AKT, PTEN, Sonic Hedgehog, TGF-beta2, VEGF, Wnt/beta-catenin; Other pathways: Cell Cycle: G1/S Checkpoint Regulation, Cell Cycle: G2/M DNA, Damage Checkpoint Regulation, Nucleotide Excision Repair Pathway, Protein Ubiquitination Pathway.
(1.87 MB TIF)

Figure S4 Progressive chemo-resistance and tumorigenicity in GL26. (A) Cell numbers + indicated concentrations of temozolamide were determined for low-passage (<5) GL26nu, GL26B6 and GL26B6V using a Coulter counter, and demonstrates progressive chemo-resistance related to anti-tumor T cell response strength. (B) Female nude mice (Harlan, Inc.; right panel) as indicated were each injected intracranially with 100 GL26 (n = 5), GL26B6 (n = 5), or GL26B6V (n = 5) tumor cells, and days to survival assessed by log-rank statistics. GL26-implanted mice survived marginally but significantly longer than GL26B6V-implanted mice only at doses of 25 cells (P = 0.049; not shown). (C, D) Fifty-thousand GL26B6-GFP or parental GL26 cells were implanted into female hosts, with significantly longer survival of GL26B6-GFP-bearing nudes (Harlan, Inc.) and significantly shorter survival of GL26B6-GFP-bearing WT C57Bl/6 (Jackson Labs; black) confirmed relative to GL26-bearing females prior to further analysis (P = 0.004 and P = 0.029, respectively). Fifty-thousand total tumor cells were then implanted into nude or WT brains at the indicated ratios of admixed GL26B6-GFP (GFP+) to parental GL26 cells, and brains from terminally symptomatic mice either sectioned for serial H&E/immunofluorescence analysis (C, right and left panels, respectively), or tumors excised, recovered by adherence to tissue culture plastic, and numbers of total and GFP+ tumor cells counted separately (n = 2 for C57BL/6; n = 3 for nude mice). Average proportions of GFP+ tumor cells + standard error are depicted in (D). GFP+ and GFP- tumors were present in all mice (C), in expected proportions at 4:1 GL26B6-GFP:GL26 in nude or WT (2- to 8-fold GFP+; P = 0.222 between nude and WT, 2-sided T test), with GL26B6-GFP overrepresentation at 1:4 in WT relative to nude (1.9 vs., 0.7-fold GFP+; P = 0.00032, 2-sided T test).
(1.96 MB TIF)

Table S1 Probesets for human GBM analysis. Entire lists of 1581 vaccine-altered, 202 EGFR- and SHH-related, 163 cytokine/immune modulator, and 944 progenitor cell/differentiation probesets (HG133+2 chip) used in microarray analyses in Figures 1, 6, S1, S2, S3, S4, and S6, can be found in Table S1.
(0.13 MB XLS)

Table S2 Probesets for mouse GL26 analysis. Entire lists of 1293 vaccine-altered, 158 EGFR- and SHH-related, 136 cytokine/immune modulator, and 763 progenitor cell/differentiation probesets (MG480+2 chip) used in microarray analyses in Figures 1, 6, S1, S2, S3, S4, and S6, can be found in Table S2.
(0.11 MB XLS)
