# Supplementary figures and images for: T Cells Enhance Stem-Like Properties and Conditional Malignancy in Gliomas
Source: PLoS One. 2010 Jun 7;5(6):e10974. doi: 10.1371/journal.pone.0010974 (PMC2881867; doi:10.1371/journal.pone.0010974)

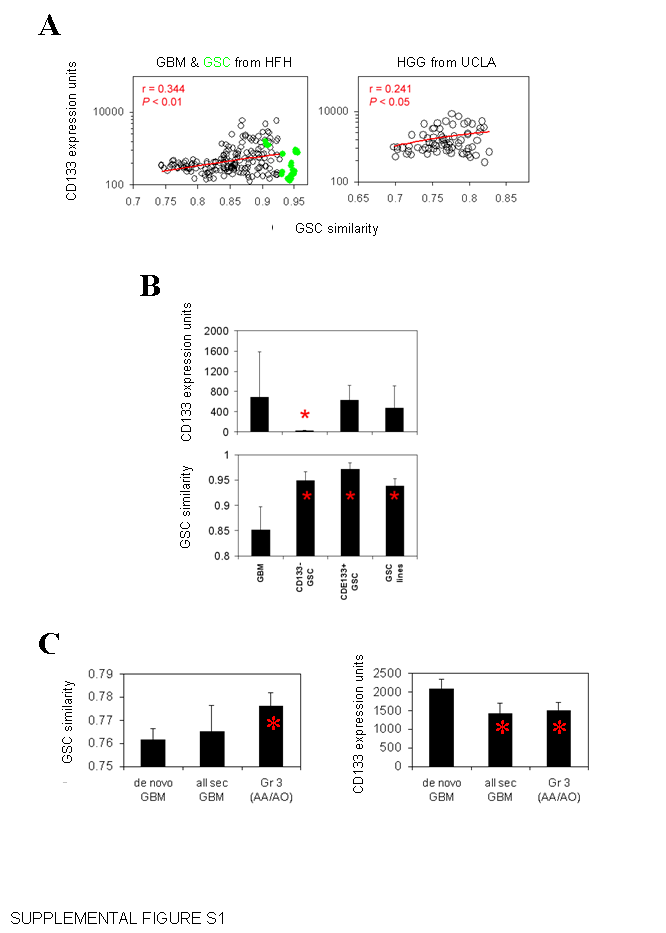

Supplement: Figure S1 — Validation of GSC similarity to distinguish CSCs from non-fractionated GBM. (A) GSC similarity (Pearson's coefficient for similarity to GSCs across all transcripts), and CD133 expression, were determined for GBM from HFH (n = 200) and high-grade gliomas from UCLA (n = 45; GEO accession #GSE4412), plotted against each other for each individual sample, trendlines generated, and r and P values determined as depicted. CSC similarity correlated significantly with CD133 expression within two separate databases. (B) Ability of CD133 expression, or GSC similarity, to distinguish non-fractionated GBM from CD133− or CD133+ GSCs (29) (GEO accession #GDS2728) or from stem cell media-cultured GBM lines from 2 patients (37) (GEO accession #GSE4536); was determined (P<0.01 denoted by red asterisk). Unlike CD133 expression, only GSC similarity distinguished CD133+ or CD133 GSCs (from multiple sources) from surgical GBM samples (C) GSC similarity (Pearson's coefficient for similarity to GSCs - GEO accession #GDS2728 - across all transcripts), and CD133 expression, were determined for de novo GBM, secondary GBM, and grade 3 gliomas from UCLA (GEO accession #GSE4412) and each parameter assessed for inter-group differences by one-tailed T-test (P<0.01 denoted by red asterisk). CD133 expression has been shown to be highest in de novo GBM (29), and this was arguably the case in the samples we analyzed, but global GSC similarity was highest in grade 3 gliomas and secondary GBM (Fig. S1C). Global GSC similarity thus paralleled increased numbers of CSCs reported for lower-grade brain tumors (1, 2). These data validated that gene expression similarity distinguishes stem-like gliomas more faithfully than does CD133 expression. (1.87 MB TIF) [file pone.0010974.s001.tif]

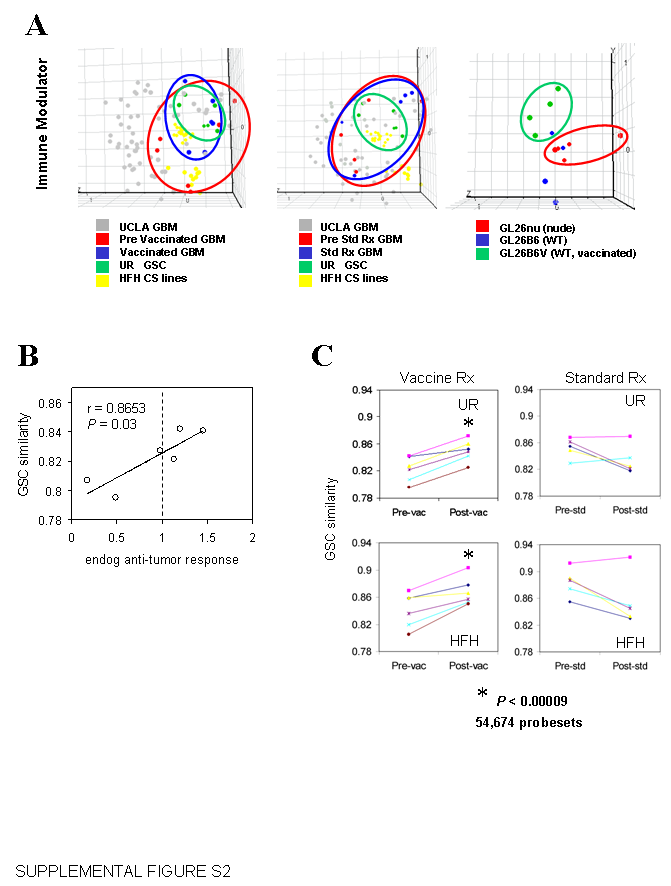

Supplement: Figure S2 — Stem-like gene expression accompanies endogenous and vaccine-induced anti-tumor T cell activity in gliomas. (A) Principal Component Analyses focused on discrete gene lists were plotted in GenespringGX7.3, and group clusters circled, on the following: 59 GBMs from UCLA database (“UCLA GBM”), 12 GBMs from 6 patients collected before and after DC vaccination (“vaccinated GBM”); 10 GBMs from 5 patients collected before and after standard radiation and/or chemotherapy (“control GBM”) (red); CD133 and CD133+ CSCs from 6 University of Regensberg GBM patients (29) (“UR GSC”) (green); stem cell media-cultured lines from 2 Henry Ford Hospital GBM patients (“HFH CS lines”); murine GL26 glioma samples recovered and cultured <8 passages from brains of 5 nude (GL26nu), 4 C57BL/6J (GL26B6) and 4 C57BL/6J mice vaccinated with 107 tumor lysate-pulsed DC2.4 cells 3 and 7 d post-tumor implantation (GL26B6V). Post-vaccine GBM uniquely exhibited co-clustering (relatedness) with UCLA glioma progenitors within genes involved in immune modulation (left, middle panels). GL26B6V exhibited parallel clustering in the analogous mouse gene list (right panel). (B) CSC similarity (Pearson's coefficient for similarity to GSCs across all transcripts) of GBM patients for whom IFN-γ anti-tumor response data was known prior to vaccine therapy was plotted against pre-vaccine anti-tumor response levels as described (23) revealing a significant direct correlation between CSC similarity and response magnitude. (C) Matched microarray data from the 12 vaccinated GBM (Vaccine Rx), and the 10 control GBM (Standard Rx) were assessed for similarity to averaged expression values of UR GSCs (29) (“UR”), or to 20 HFH CS lines (“HFH”), and Pearson's coefficients across 54,674 transcripts plotted for each patient in line plots. Significantly increased stem-like gene expression (asterisks) was unique to post-vaccine samples (P<0.001; one-tailed T-test). See Fig. 1 legend for GEO accession numbers. (1.79 MB TIF) [file pone.0010974.s002.tif]

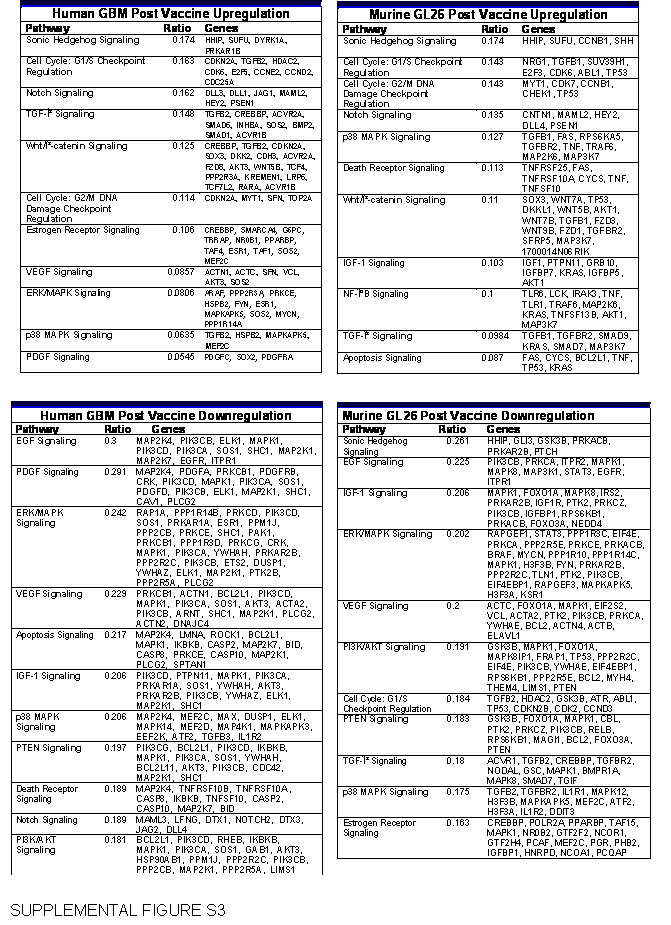

Supplement: Figure S3 — Ingenuity Pathways Analysis of vaccine-altered glioma genes. The involvement of vaccine-altered genes in 20 pathways containing known oncogenes is scored by a ratio of the number of vaccine-altered genes to the number of genes in each pathway. (A) Human GBM post-vaccine up-regulation. (B) Murine GL26 post-vaccine up-regulation. (C) Human GBM post-vaccine down-regulation. (D) Murine GL26 post-vaccine down-regulation. Up and down regulated genes were scored separately. Shown here are the 11 pathways with the highest ratios of changed genes to total genes among 20 pathways of interest. Signaling Pathways: Apoptosis, Death Receptor, EGF, ERK/MAPK, Estrogen Receptor, GF-1, NF-kappaB, Notch, p38 MAPK, PDGF, PI3K/AKT, PTEN, Sonic Hedgehog, TGF-beta2, VEGF, Wnt/beta-catenin; Other pathways: Cell Cycle: G1/S Checkpoint Regulation, Cell Cycle: G2/M DNA, Damage Checkpoint Regulation, Nucleotide Excision Repair Pathway, Protein Ubiquitination Pathway. (1.87 MB TIF) [file pone.0010974.s003.tif]

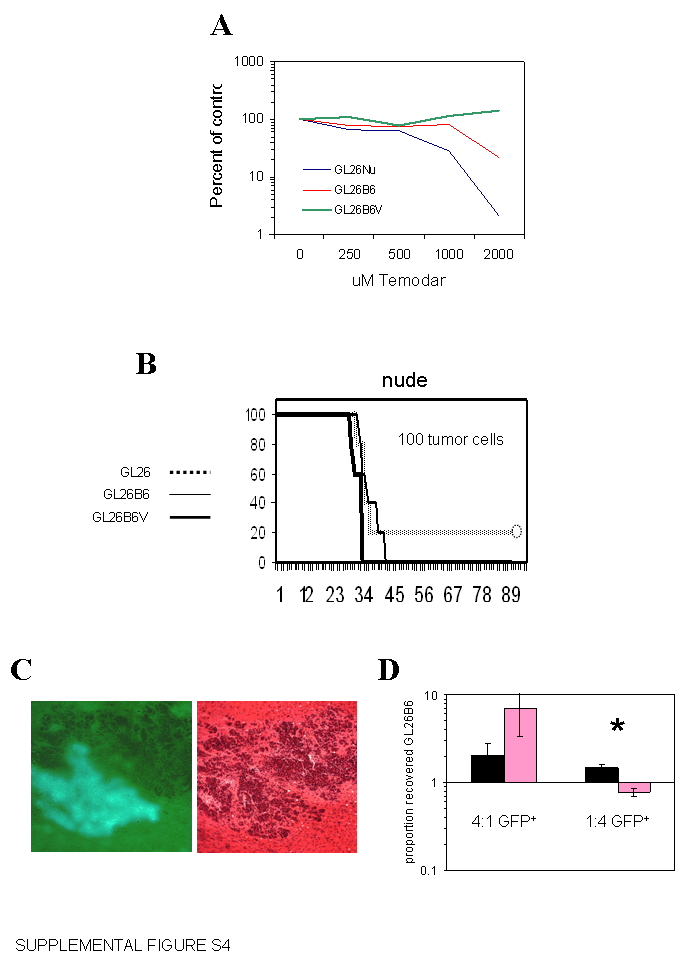

Supplement: Figure S4 — Progressive chemo-resistance and tumorigenicity in GL26. (A) Cell numbers + indicated concentrations of temozolamide were determined for low-passage (<5) GL26nu, GL26B6 and GL26B6V using a Coulter counter, and demonstrates progressive chemo-resistance related to anti-tumor T cell response strength. (B) Female nude mice (Harlan, Inc.; right panel) as indicated were each injected intracranially with 100 GL26 (n = 5), GL26B6 (n = 5), or GL26B6V (n = 5) tumor cells, and days to survival assessed by log-rank statistics. GL26-implanted mice survived marginally but significantly longer than GL26B6V-implanted mice only at doses of 25 cells (P = 0.049; not shown). (C, D) Fifty-thousand GL26B6-GFP or parental GL26 cells were implanted into female hosts, with significantly longer survival of GL26B6-GFP-bearing nudes (Harlan, Inc.) and significantly shorter survival of GL26B6-GFP-bearing WT C57Bl/6 (Jackson Labs; black) confirmed relative to GL26-bearing females prior to further analysis (P = 0.004 and P = 0.029, respectively). Fifty-thousand total tumor cells were then implanted into nude or WT brains at the indicated ratios of admixed GL26B6-GFP (GFP+) to parental GL26 cells, and brains from terminally symptomatic mice either sectioned for serial H&E/immunofluorescence analysis (C, right and left panels, respectively), or tumors excised, recovered by adherence to tissue culture plastic, and numbers of total and GFP+ tumor cells counted separately (n = 2 for C57BL/6; n = 3 for nude mice). Average proportions of GFP+ tumor cells + standard error are depicted in (D). GFP+ and GFP- tumors were present in all mice (C), in expected proportions at 4:1 GL26B6-GFP:GL26 in nude or WT (2- to 8-fold GFP+; P = 0.222 between nude and WT, 2-sided T test), with GL26B6-GFP overrepresentation at 1:4 in WT relative to nude (1.9 vs., 0.7-fold GFP+; P = 0.00032, 2-sided T test). (1.96 MB TIF) [file pone.0010974.s004.tif]
